# Axillary Reactive Lymphoid Hyperplasia, Likely Due to Unicentric Castleman Disease, and the Concurrent Presence of Orbital Mucosa-Associated Lymphoid Tissue (MALT) Lymphoma: A Six-Year Follow-Up Study

**DOI:** 10.7759/cureus.73775

**Published:** 2024-11-15

**Authors:** Toshihiko Matsuo, Takehiro Tanaka, Tomokazu Fuji, Daisuke Ennishi

**Affiliations:** 1 Department of Ophthalmology, Graduate School of Interdisciplinary Science and Engineering in Health Systems, Okayama University, Okayama, JPN; 2 Department of Opthalmology, Okayama University Hospital, Okayama, JPN; 3 Department of Pathology, Graduate School of Medicine, Dentistry, and Pharmaceutical Sciences, Okayama University, Okayama, JPN; 4 Department of Gastroenterological Surgery, Graduate School of Medicine, Dentistry, and Pharmaceutical Sciences, Okayama University, Okayama, JPN; 5 Department of Hematology and Oncology, Center for Comprehensive Genomic Medicine, Okayama University Hospital, Okayama, JPN

**Keywords:** blepharoptosis, castleman disease, extranodal marginal zone b-cell lymphoma of mucosa-associated lymphoid tissue (malt) lymphoma, pancreatic cancer, radiation, reactive lymphoid hyperplasia

## Abstract

Castleman disease is a lymphadenopathy of unknown cause at a single site, which is designated as unicentric Castleman disease, or at multiple sites designated as multicentric Castleman disease. We present a patient who showed axillary reactive lymphoid hyperplasia, likely due to unicentric Castleman disease, and orbital extranodal marginal zone B-cell lymphoma of mucosa-associated lymphoid tissue (MALT) lymphoma in a six-year follow-up. A 76-year-old man had a painless left axillary mass for an unknown period and also left complete blepharoptosis with no other systemic symptoms. Suspected of lymphoma, iliac bone marrow biopsy showed no anomalous cells, and positron emission tomography demonstrated abnormal uptake at the left axilla and in the left superior anterior orbit. Incisional biopsy of the left axillary mass demonstrated hyperplastic lymphoid follicles with an atrophic germinal center and prominent small vessels in the follicular center, indicative of unicentric Castleman disease. One year later, annual follow-up positron emission tomography disclosed a high uptake site, next to the previously-identified cyst, in the pancreatic body. Trans-gastric fine needle pancreatic biopsy proved adenocarcinoma and he underwent subtotal stomach-preserving pancreaticoduodenectomy with jejunal anastomosis. He was well for six months after the surgery and thus, underwent resection of the left orbital lesion at 78 years old. The pathology of the orbital lesion showed ambiguous nodular structure with massive infiltration with CD20-positive medium-sized lymphoid cells which were κ monotype in immunoglobulin light chain restriction, indicative of MALT lymphoma. In the four-year period of the COVID-19 pandemic, he was healthy and followed with no treatment until the age of 82 years when he underwent radiation (46 Gy) to the left axillary lesion which did not regress. He then underwent eyelid levator muscle plication for left blepharoptosis since the left orbital lesion remained unpalpable. The six-year follow-up showed that concurrent and independent orbital MALT lymphoma and axillary reactive lymphoid hyperplasia, likely due to unicentric Castleman disease, were both stable. The present case illustrates how important it is to make pathological diagnoses in different anatomical lesions after the initial diagnosis of Castleman disease.

## Introduction

Castleman disease is a lymphadenopathy of unknown cause at a single site, which is designated as unicentric Castleman disease, or at multiple sites designated as multicentric Castleman disease [1-3]. The disease is usually detected incidentally by imaging such as plain chest X-ray, computed tomography scan, and magnetic resonance imaging. Predominant locations involved with unicentric Castleman disease are the mediastinal region, abdomen, and retroperitoneal space [4]. The current diagnostic criteria for Castleman disease are based on the histopathological diagnosis and the exclusion of other diseases with lymphadenopathy which include lymphoma, infectious lymphadenitis, and autoimmune diseases. The key pathological features are hyperplastic lymphoid follicles which are associated with an atrophic germinal center and thickened mantle zone [4,5]. In the spectrum of additional histopathological features, vascular proliferation with varying levels of hyalinization is observed inside the follicles, indicative of hyaline vascular type at one end. Plasma cells are infiltrated mainly in interfollicular spaces, indicative of plasmacytic type at the other end.

In this study, we present an old adult who showed a painless large axillary mass, suggestive of lymphadenopathy, in association with no systemic symptoms but with unilateral complete blepharoptosis in the first place. The biopsy of the axillary lesion proved reactive lymphoid hyperplasia, likely due to unicentric Castleman disease, while an excisional biopsy of the orbital mass lesion as a cause of blepharoptosis was postponed because the patient was found to have pancreatic cancer. After successful pancreaticoduodenectomy with reconstruction, later resection of the orbital lesion proved extranodal marginal zone B-cell lymphoma of mucosa-associated lymphoid tissue (MALT) lymphoma.

## Case presentation

**Figure 1:**
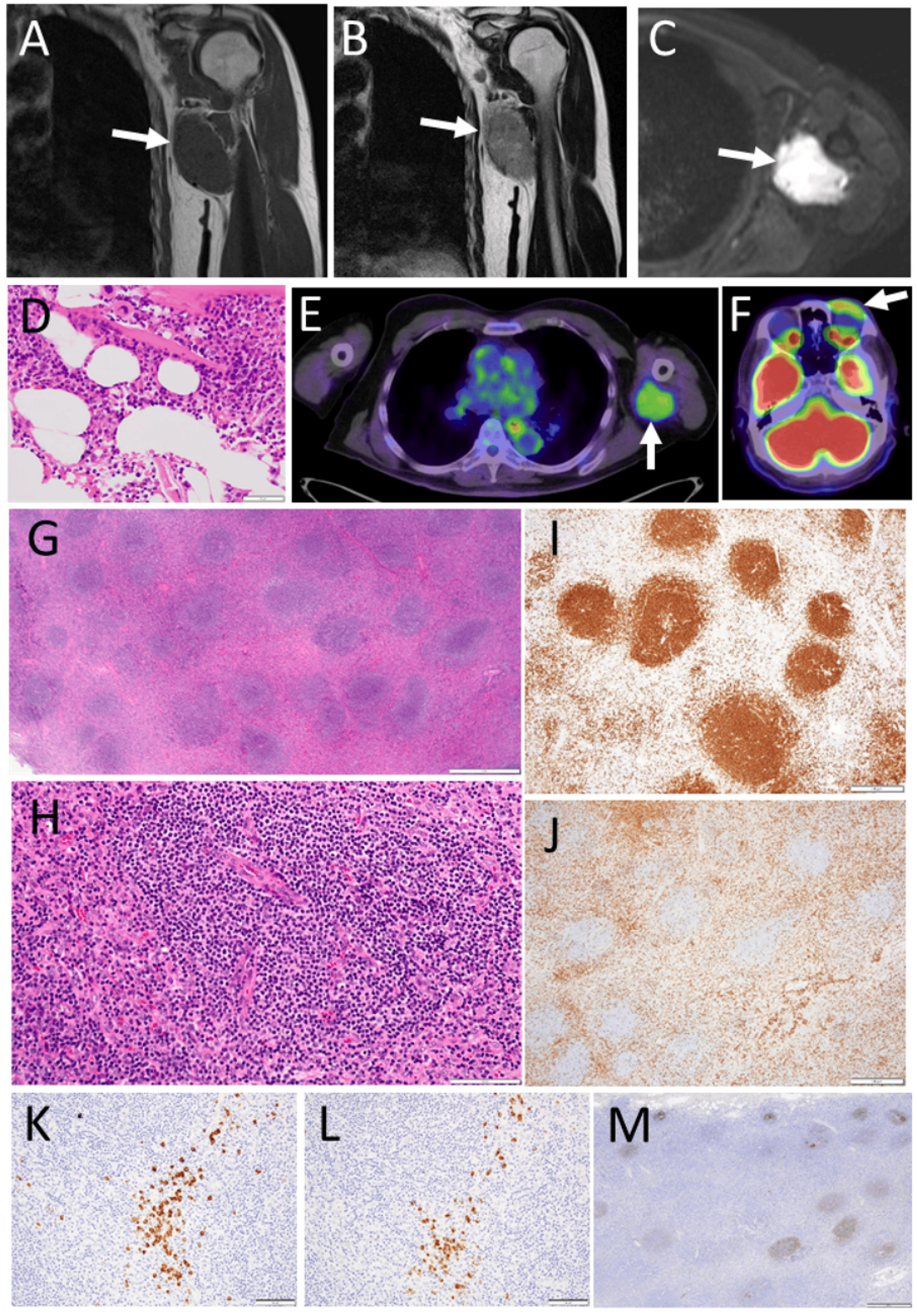
Left axillary mass. Magnetic resonance imaging at the age of 76 years, showing left axillary mass (arrows) in T1-weighted (A) and T2-weighted (B) coronal images and diffusion-weighted axial image (C). Iliac bone marrow biopsy (D), showing normocellular marrow with no anomalous cells. Positron emission tomography with axial computed tomography scans, showing high uptake (arrows) at left axillary mass with SUVmax at 3.51 (E) and in left superior anterior orbit (F). Incisional biopsy of left axillary mass, leading to the diagnosis of Castleman disease with hyaline vascular type at the age of 76 years. Hyperplasia of lymphoid follicles (G) with atrophic germinal center and dominant mantle layer. Prominent small vessels in follicular center (H). CD20-positive B-lymphocytes (I) and CD3-positive T-lymphocytes (J) segregate from each other. By in situ hybridization, immunoglobulin light chain κ (Igκ)-positive cells (K) and immunoglobulin light chain λ (Igλ)-positive cells (L) are equally distributed, indicating the bitype. CD21-positive follicular dendritic cells appear coarse (M). White scale bar = 50 µm in D, 1000 µm in G, 100 µm in H, K, L, 500 µm in I, J, M.

A 76-year-old man had a painless large mass at the left axilla for an unknown period. He also had left persistent complete blepharoptosis for a few years. He had been taking tadalafil 5 mg and dutasteride 0.5 mg daily for benign prostatic hyperplasia, amlodipine 2.5 mg and a combination tablet of valsartan 80 mg and hydrochlorothiazide 6.25 mg for hypertension, and febuxostat 10 mg for hyperuricemia for a long term. He did not have any other past history and did not smoke or drink alcohol. He was referred to an oncologist, suspected of lymphoma. He was healthy and had neither fever, night sweats, or weight loss. Physical examinations revealed nothing to be noted except for a left axillary well-defined, movable, elastic-hard mass with 7 cm diameter and left complete blepharoptosis. Eye movement had no limitation in both eyes. Magnetic resonance imaging showed a well-defined mass at the left axilla which showed the same signal with the muscle in a T1-weighted image (Figure 1A), but a different signal in a T2-weighted image (Figure 1B), and a high signal in a diffusion-weighted image (Figure 1C). Suspected of lymphoma, iliac bone marrow biopsy (Figure 1D) showed the normocellular marrow with no anomalous cells. Positron emission tomography demonstrated a high uptake (maximum standardized uptake value (SUVmax)=3.51) at the left axilla (Figure 1E) and also in the left superior anterior orbit (Figure 1F). As a diagnostic procedure, an incisional biopsy of the left axillary mass demonstrated hyperplastic lymphoid follicles (Figure 1G) with an atrophic germinal center and prominent small vessels in the follicular center (Figure 1H), indicative of unicentric Castleman disease. Immunohistochemically, CD20-positive B-cells (Figure 1I) and CD3-positive T-cells (Figure 1J) were segregated from each other and immunoglobulin light chain restriction was not noted (Figures 1K, 1L). CD21-positive follicular dendritic cells appeared coarse (Figure 1M). There were several immunoglobulin G4 (IgG4)-positive cells but the number of IgG4-positive cells did not fulfill the criteria for IgG4-related disease. The patient was followed with no treatment since he had no systemic symptoms and did not wish for additional treatment.

**Figure 2:**
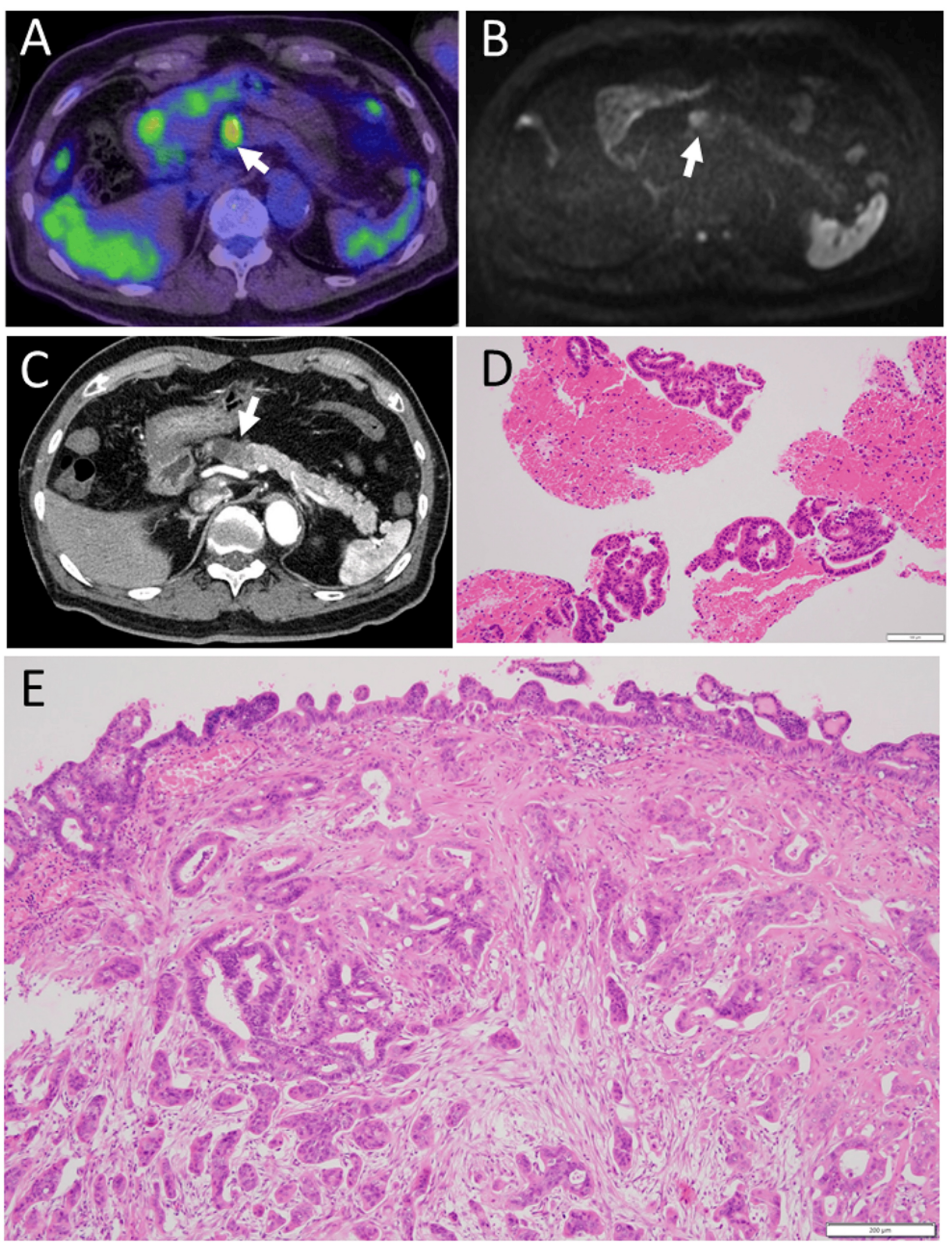
Pancreatic lesion. Positron emission tomography, one year later at 77 years old, showing abnormal uptake (arrow) in pancreatic body (A). Magnetic resonance imaging just before trans-gastric pancreatic biopsy, showing high signal of pancreatic head to body (arrow) in diffusion-weighted image (B). Computed tomography scan with enhancement at the age of 78 years just before pancreaticoduodenectomy, showing a low-enhanced mass with a cyst (arrow) in the pancreatic head to body (C). Trans-gastric fine needle pancreatic biopsy (D), showing adenocarcinoma. Subtotal stomach-preserving pancreaticoduodenectomy with jejunostomy (E), showing intraductal papillary mucinous carcinoma with interstitial invasion. White scale bar =100 µm in D, 200 µm in E.

One year later at the age of 77 years, annual follow-up positron emission tomography disclosed a high uptake site, next to the previously-identified cyst, in the pancreatic body (Figure 2A), in addition to uptake in the left axilla (SUVmax=3.4) and the left orbit which had been present a year previously (Figures 1E, 1F). The pancreatic body lesion with a diameter of 1.7 cm showed a high signal in a diffusion-weighted image of magnetic resonance imaging (Figure 2B) and demonstrated initial low perfusion with a gradual increase of staining by computed tomography scan with contrast enhancement (Figure 2C). Trans-gastric fine needle pancreatic biopsy proved adenocarcinoma (Figure 2D).

**Figure 3:**
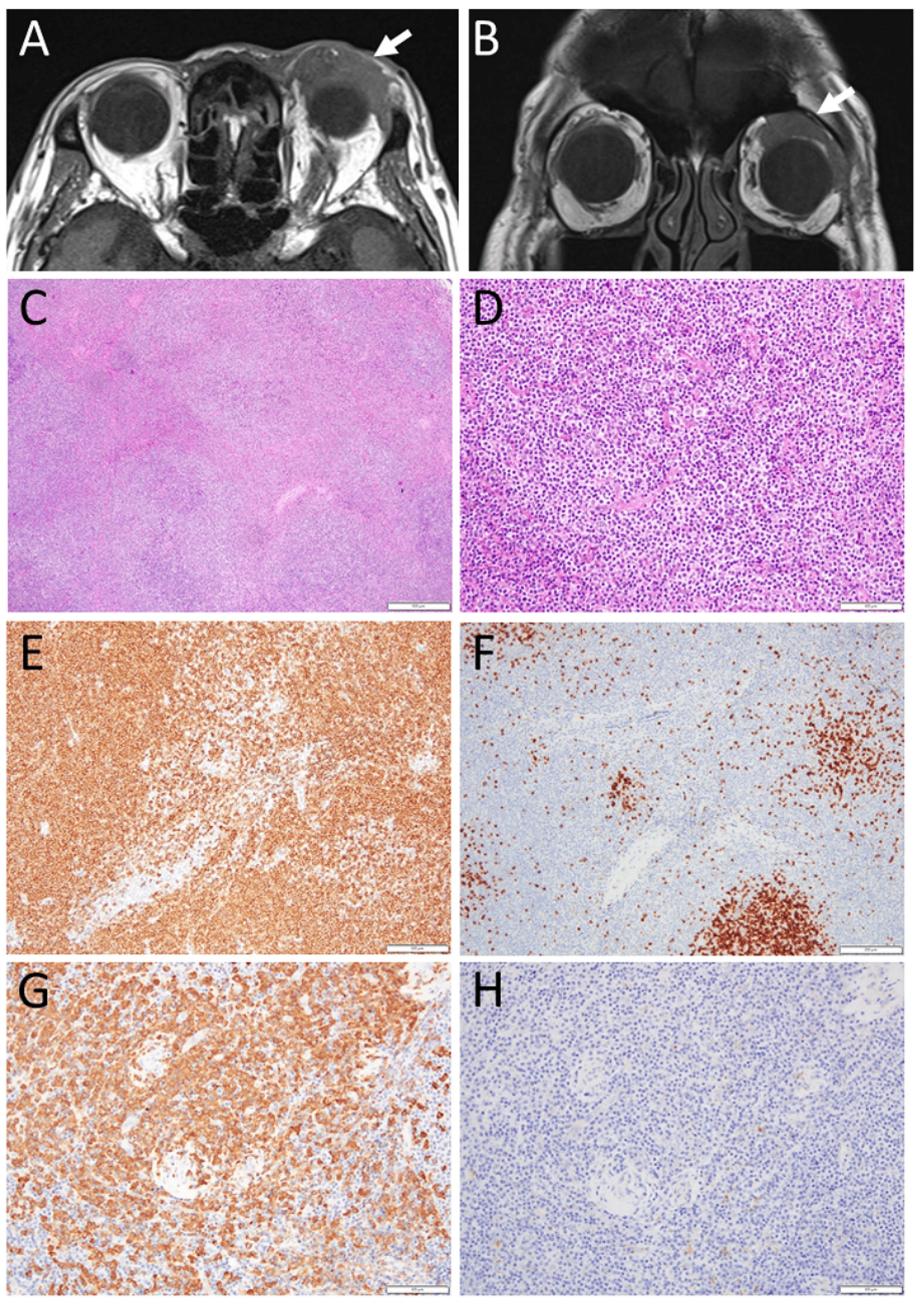
Left orbital mass lesion. Magnetic resonance imaging at the age of 77 years, showing a left superior anterior orbital mass (arrows) in T1-weighted axial (A) and coronal (B) images. Resection of left orbital mass, leading to the diagnosis of extranodal marginal zone B-cell lymphoma of mucosa-associated lymphoid tissue (MALT lymphoma) at the age of 78 years. Ambiguous nodular structure (C) with massive infiltration with medium-sized lymphoid cells (D). Most of lymphoid cells are positive for CD20 (E), admixed with small foci of CD3-positive cells (F). By in situ hybridization, lymphoid cells are all positive for immunoglobulin light chain κ (Igκ, G), and all negative for immunoglobulin light chain λ (Igλ, H), indicating immunoglobulin light chain restriction to κ (κ monotype). White scale bar = 500 µm in C, 100 µm in D, G, H, 200 µm in E, F.

At that time, head magnetic resonance imaging showed a mass in the superior and anterior orbit on the left side (Figures 3A, 3B). The left orbital mass resection was postponed to give priority to pancreatic cancer surgery. At 78 years, he underwent subtotal stomach-preserving pancreaticoduodenectomy with reconstruction by jejunal anastomosis (SSPPD-IIA-1). The pathology of the resected pancreas revealed intraductal papillary mucinous carcinoma with interstitial invasion (Figure 2E).

He was well for six months after the pancreatic surgery, and thus, underwent resection of the left orbital lesion at the age of 78 years. Head magnetic resonance imaging just before the surgery showed a stable left orbital mass. The pathology of the orbital lesion showed ambiguous nodular structure (Figure 3C) with massive infiltration with medium-sized lymphoid cells (Figure 3D) that were positive for CD20 (Figure 3E), admixed with CD3-positive cells (Figure 3F), and that was κ monotype in immunoglobulin light chain restriction (Figures 3G, 3H), indicative of extranodal marginal zone B-cell lymphoma of MALT lymphoma. IgG4-positive cells were totally absent. In the four-year period of the COVID-19 pandemic, he was well and followed with no treatment until the age of 82 years when he underwent radiation (46 Gy in total with 23 fractions) to the left axillary lesion which had not been regressing. He then underwent eyelid levator muscle plication for the persistent left blepharoptosis at local anesthesia since the left orbital lesion remained unpalpable. At the latest visit, he showed mild cataracts in both eyes, and his best-corrected visual acuity in decimals was 1.0 in the right eye and 0.6 in the left eye with no diplopia. He still had the left axillary mass in the diameter of 5 cm which had been reduced to 70% size in half a year after the radiation.

## Discussion

The present patient is unique in the point that he appeared to concurrently and independently have the left axillary mass and left orbital mass which were pathologically diagnosed as reactive lymphoid hyperplasia, likely due to unicentric Castleman disease, and MALT lymphoma, respectively. In contrast with the axillary unicentric Castleman disease which was diagnosed at first, the orbital MALT lymphoma was diagnosed two years later because the patient underwent surgery for incidentally detected pancreatic cancer. The patient was followed up with no additional treatment for six years in total from the initial visit mainly because of the COVID-19 pandemic. In this six-year period, the axillary mass remained unchanged and the orbital lesion did not relapse even though the resection left behind a small residual lesion in the orbit. He was healthy and ambulatory after the pancreatic surgery. According to his wish, he finally underwent radiation toward the left axillary mass and eyelid levator muscle plication for the persistent complete blepharoptosis. To summarize the course, the patient showed stable axillary unicentric Castleman disease in six years with no treatment and stable residual lesion of orbital MALT lymphoma four years after the resection. He did not have other symptoms such as fever, weight loss, and general fatigue throughout the course.

The axillary large mass in this patient would be diagnosed pathologically as reactive lymphoid hyperplasia in a broad sense. The terminology of “reactive lymphoid hyperplasia” means “lymphoid hyperplasia of unknown cause” and does not indicate “lymphoid hyperplasia reactive to other diseases”. The pathological findings such as the atrophic germinal center and prominent small vessels in the follicular center were most consistent with Castleman disease. Indeed, reactive lymphoid hyperplasia is a mixture of various diseases, including Castleman disease with an unknown cause of inflammation [6]. After the diagnosis of Castleman disease by incisional biopsy of the axillary mass, the left orbital lesion was suspected to be part of Castleman disease even though orbital involvement with Castleman disease has not been known. In the meantime, a pancreatic lesion detected by imaging was confirmed as pancreatic cancer by biopsy. The priority was given to pancreatectomy and the resection of the orbital mass was postponed. The persistent complete blepharoptosis on the left side did not change in the course of six years and would be related to the orbital mass lesion.

There have been several reports until now which describe multicentric and unicentric Castleman disease in association with different types of lymphoma such as Hodgkin lymphoma, B-cell lymphoma, and T-cell lymphoma [7-11]. In reported cases of unicentric Castleman disease in association with lymphoma, mass lesions diagnosed as Castleman disease involved cervical, inguinal, and axillary lymph nodes in addition to well-known mediastinal and retroperitoneal lymph nodes [7-11]. Of these, two cases were reported to develop unicentric Castleman disease and lymphoma concurrently in different anatomical locations [7]. In the present patient, reactive lymphoid hyperplasia, likely due to unicentric Castleman disease, and MALT lymphoma in different anatomical locations would be simply combined by chance but raises the possibility of a shared underlying pathophysiology in the two diseases. As a potential link between Castleman disease and lymphoma development, Castleman disease might serve as the background for the development of lymphoma, based on pathological findings in some cases that lymphoma lesions contained Castleman disease-like features [12-14]. Further research needs to be done to evaluate the common risk factors for Castleman disease and MALT lymphoma.

In the present patient who at first had a pathological diagnosis of axillary Castleman disease, the pancreatic lesion and orbital mass lesion might be misdiagnosed as part of Castleman disease but were correctly diagnosed as such by pathological examinations. Positron emission tomography was repeated a year later in this patient because Castleman disease is known to be associated with lymphoma. Indeed, Castleman disease which occurs rarely in the pancreas may be misdiagnosed as pancreatic cancer [15,16] or neuroendocrine tumor [17]. In the present patient, the pancreatic lesion which was detected first by positron emission tomography was evaluated in detail by magnetic resonance imaging and computed tomography with enhancement, leading to the diagnosis of pancreatic cancer by trans-gastric needle biopsy.

It should be noted that the left orbital MALT lymphoma was stable two years before the diagnosis by the resection and also did not show relapse from the residual lesion four years after the resection. The orbit with ocular adnexa is a common site for MALT lymphoma [18] and orbital mass lesions should be differentially diagnosed by pathological examinations from inflammatory lesions such as reactive lymphoid hyperplasia and IgG4-related disease [19,20]. The residual lesion of MALT lymphoma is usually treated by local radiation or systemic chemotherapy such as rituximab monotherapy. The present patient did not wish to have additional treatment for orbital MALT lymphoma since he did not have systemic manifestations of lymphoma. He was followed every 4 months since the recurrence rate of Castleman disease remains to be elucidated.

## Conclusions

The present patient had axillary reactive lymphoid hyperplasia, likely due to unicentric Castleman disease, and the concurrent presence of orbital MALT lymphoma. After the pathological diagnosis by incisional biopsy of the axillary lesion and resection of the orbital lesion, respectively, both lesions were stable in six years of follow-up. Unicentric Castleman disease and MALT lymphoma might be combined by chance in this patient or might share a common background which remains unknown. The course of this patient illustrates how important it is to make pathological diagnoses in different anatomical lesions after the initial pathological diagnosis of Castleman disease.
